# AGING IN PLACE IN JAPAN: CHALLENGES AND FACILITATORS OF OLDER-TO-OLDER CAREGIVING

**DOI:** 10.1093/geroni/igad104.2892

**Published:** 2023-12-21

**Authors:** Mineko Wada, Hideaki Hanaoka

**Affiliations:** Hiroshima University, Hiroshima, Hiroshima, Japan; Hiroshima University, Hiroshima, Hiroshima, Japan

## Abstract

The proportion of older adults (age 65+) in Japan has been steadily growing, and reached almost 30% in 2022, its highest-ever level. As the benefits of aging in place have been promoted at individual, community, and policy levels, the number of households where an older adult cares for another older adult has been increasing. However, currently little is known about the challenges and facilitators of older-to-older caregiving. This study aims to review extant literature to identify challenges experienced by older-to-older caregivers and facilitators that could help them. Drawing on a review protocol informed by Arksey and O’Malley’s framework and Preferred Reporting Items for Systematic reviews and Meta-Analysis extension for Scoping Reviews (PRISMA-ScR), we searched six databases (MEDLINE, CINAHL, PsycINFO, Cochrane, Scopus, Google Scholar) using combinations of search terms including “older,” “aged,” “senior,” “caregiver,” “carer,” “care provider,” and “care recipient.” To be included in the study, the literature must be written in English and based on empirical studies focusing on older caregivers (age 65+) who care for older adults (age 65+) in community settings. The reviewed literature suggests that older caregivers experience substantial distress, caregiver burden, and sleep deprivation, which increases the risk of depression and diminished well-being, particularly if they care for older adults living with dementia. Facilitators of caregiving include strategies for self-care and problem-solving to address challenges. Future research needs to investigate (1) support options that could reduce older caregivers’ burden and promote their well-being and (2) older care recipients’ experiences of being cared for by older cares.

